# Residential urban food environment profiles and diet outcomes among adults in Brooklyn, New York: a cross-sectional study

**DOI:** 10.1017/S1368980022002476

**Published:** 2023-04

**Authors:** Roger Figueroa, Katherine Baker, Joel Capellan, Laura C Pinheiro, Laura Burd, Jane Lim, Reah Chiong, Relicious Eboh, Erica Phillips

**Affiliations:** 1 Division of Nutritional Sciences, College of Human Ecology, Cornell University, 244 Garden Avenue, Ithaca, NY 14853, USA; 2 Law & Justice Studies, Rowan University, 215 Mullica Road, Glassboro, NJ 08028, USA; 3 Division of General Internal Medicine, Department of Medicine, Cornell Center for Health Equity, Weill Cornell Medicine College, 338 East 66th Street, New York, NY 10065, USA; 4 Master of Public Health, College of Veterinary Medicine, Cornell University, 602 Tower Road, Ithaca, NY 14853, USA

**Keywords:** Diet, Latent profile analysis, GIS, Residential urban food environment, Adults

## Abstract

**Objective:**

To assess the clustering properties of residential urban food environment indicators across neighbourhoods and to determine if clustering profiles are associated with diet outcomes among adults in Brooklyn, New York.

**Design:**

Cross-sectional.

**Setting:**

Five neighbourhoods in Brooklyn, New York.

**Participants:**

Survey data (*n* 1493) were collected among adults in Brooklyn, New York between April 2019 and September 2019. Data for food environment indicators (fast-food restaurants, bodegas, supermarkets, farmer’s markets, community kitchens, Supplemental Nutrition Assistance Program application centres, food pantries) were drawn from New York databases. Latent profile analysis (LPA) was used to identify individuals’ food access-related profiles, based on food environments measured by the availability of each outlet within each participant’s 800-m buffer. Profile memberships were associated with dietary outcomes using mixed linear regression.

**Results:**

LPA identified four residential urban food environment profiles (with significant high clusters ranging from 17 to 57 across profiles): limited/low food access, (*n* 587), bodega-dense (*n* 140), food swamp (*n* 254) and high food access (*n* 512) profiles. Diet outcomes were not statistically different across identified profiles. Only participants in the limited/low food access profile were more likely to consume sugar-sweetened beverages (SSB) than those in the bodega-dense profile (b = 0·44, *P* < 0·05) in adjusted models.

**Conclusions:**

Individuals in limited and low food access neighbourhoods are vulnerable to consuming significant amounts of SSB compared with those in bodega-dense communities. Further research is warranted to elucidate strategies to improve fruit and vegetable consumption while reducing SSB intake within residential urban food environments.

Diet-related chronic health conditions (i.e. obesity, Type II diabetes, CVD and cancer) are among the leading causes of death in the USA. These conditions disproportionately impact racial and ethnic minoritised adults and those of low socio-economic status^(1–4)^. Dietary intake is one of the main precursors (or risk factors) associated with diet-related chronic diseases^(5)^, which is influenced by the food environment^(6)^. The food environment within urban settings can significantly differ in its degree of accessibility, affordability and availability of nutrient-dense foods that are thought to be protective against the aforementioned health conditions. This variability between food environments often tracks the degree of poverty and racial/ethnic composition of each neighbourhood^(7)^.

In major cities within high-income countries, most studies from the last 10–15 years have adopted two primary approaches to operationalise food environments: first, density approaches quantify food outlet availability using buffer methods (i.e. Euclidean, Geodesic), and second, proximity approaches assess the distance to food retailers by measuring distance and travel times^(8)^. Studies adopting proximity approaches often assess an individual’s access to each food outlet independently with few examining multiple food retailers and their aggregate impact on dietary intake in combination^(9–12)^.

Recently, researchers have applied a ‘clustering’ approach to measure the food environment using geospatial data^(13–15)^. This novel approach attempts to shed light on food access inequities in neighbourhoods by accounting for multiple food outlets at the same time. To date, the following two broad findings have emerged from this approach. First, population density, income and race are factors related to the access and availability of healthy and unhealthy food retailers^(13)^. Second, although evidence suggests that with greater population density comes more significant number of food outlets (particularly unhealthy food retailers), socio-economic disadvantage at the sub-population level (i.e. census tract) might inhibit purchasing power by residents^(14)^. Clustering food environment indicators might prove to be a more effective way of depicting food environment variability across multiple neighbourhoods while also accounting for the socio-demographic characteristics of the residents.

A residential food environment can be defined as the number of food outlets retailers available within a given physical distance from an individual’s residence. While several studies have shown significant relationships between various residential food environments, dietary intake and multiple diet-related health outcomes, some studies have found null results when testing such relationships^(7,16,17)^. For example, residential access to and/or in proximity to select food retailers (i.e. fast-food restaurants, bodegas) is significantly associated with increased consumption of sugar-sweetened beverages (SSB), fast-food and subsequent higher rates of obesity^(18,19)^. Similarly, residential access to full-service food outlets with high availability of nutrient-dense foods (i.e. supermarkets, fruit and vegetable markets, natural food stores) is associated with an increased consumption of fruit, vegetables and water consumption^(12)^, improved diet quality (i.e. lower consumption of ultra-processed foods and SSB) and a lower BMI^(18,20,21)^. On the other hand, additional evidence suggests no significant association between the following: the neighbourhood density of fast- food restaurants and dietary quality, residential proximity to SSB retailers and actual SSB intake^(16,22,23)^ as well as supermarket availability and higher consumption of fruit and vegetables^(24)^.

In the present study, we combined density and a Euclidean buffer approach with latent profile analysis (LPA) to assess the relationship between residential urban food environment profiles and daily consumption of fruits, vegetables and SSB among adults residing in five neighbourhoods of Brooklyn, New York.

## Methods

### Data source, study sample and setting

This study used data from a cross-sectional survey designed to elucidate adults’ perception of risk for cancer and CVD and to determine if risk perception was associated with the likelihood of engaging in preventive behaviours, for example, consuming the recommended number of fruits and vegetables daily. Participants for the parent study^(25)^ were sampled from five Brooklyn neighbourhoods (Bedford-Stuyvesant, Coney Island, Crown Heights, East Flatbush and Flatbush/Midwood) where at least 20 % of the annual cancer cases were registered with the New York-Presbyterian Health Care System. Brooklyn is a borough of New York City with a population of 2·7 million residents, making it one of the most densely populated counties in the USA.^(26)^. The study inclusion criteria included adults over the age of 40 years who resided in one of the target neighbourhoods based on the zip code associated with their listed telephone number. A customised randomly ordered list of households where at least one adult (> 40 years) resided in the home and approximately half had incomes of < $34 999 was purchased from Marketing Systems Group. The median household income in Brooklyn in 2017 was $56 942, which was 17 % less than the median annual income of $68 486 across the entire state of New York. Participants contacted by telephone had the option to complete the survey via the web. Telephone calls were conducted between 2 May 22019 and 15 September 2019. The survey was conducted in English and Spanish. All participants were offered a $15 gift card for their time. The study was approved by the Institutional Review Board of Weill Cornell Medicine.

### Dependent measures

Dependent variables included one indicator for each serving of fruits, vegetables, pure fruit juice and sweetened beverage consumption. Participants responded to each outcome item on a Likert scale (Table 1). For example, the item on fruits servings asked, ‘On the days you have fruit, about how many servings do you usually eat? (A serving is generally the size of one banana, a medium apple, or a handful of grapes).’ Item response options ranged from ‘1 serving’ to ‘6 servings or more’ and also options to respond: ‘do not know’ or ‘refused’ across all items (see Table 1).

**Table 1:** Sample survey questions for participants

Survey question	Survey choice options
‘On days when you have fruit, about how many servings do you usually eat? (A serving is generally the size of one banana, a medium apple, or a handful of grapes.)’	1 serving
	2 servings
	3 servings
	4 servings
	5 servings
	6 servings or more
	Do not know
	Refused
	Not answered
‘On days when you have vegetables, about how many servings do you usually eat? (A serving is generally a half cup of cooked vegetables, or a cup of raw vegetables.)’	1 serving
	2 servings
	3 servings
	4 servings
	5 servings
	6 servings or more
	Do not know
	Refused
	Not answered
‘On days when you have sweetened beverages, about how many cups do you usually drink?’	Up to 2 cups per day
	2–4 cups per day
	More than 4 cups per day
	Do not know
	Refused
	Not answered

### Independent measures

Independent variables included seven food environment indicators measured at the community district (neighbourhood) level and included fast-food restaurants, bodegas, supermarkets, farmer’s markets, community kitchens, Supplemental Nutrition Assistance Program (SNAP) application centres and food pantries. While it is not a food retailer, SNAP application centres are considered a part of the overall food environment as it enables access to resources in order to purchase foods at eligible retailers. Fast-food restaurant data were drawn from the New York City Department of Health and Mental Hygiene (NYCDOHMH)’s online directory of restaurant inspections and included national fast-food chains (i.e. McDonald’s, Burger King, Dunkin’ Donuts, White Castle, Subway, etc.), as well as local chains that serve fast-food style food, including restaurants centred around food items like fried chicken or dollar pizza^(10)^. Food retailer data were drawn from the New York Department of Agriculture’s Division of Food Safety & Inspection dataset ‘Retail Food Stores’, which includes a listing of all retail food stores which are licensed by the Department of Agriculture and Markets^(27)^. Food retailers’ addresses including supermarkets, grocery stores and bodegas in Brooklyn, New York, were drawn from this dataset. Addresses for all Brooklyn farmer’s market data were drawn from the New York City Health map ^(28)^. Addresses of community kitchens, food pantries and SNAP application locations located in Brooklyn, New York, were drawn from the FoodHelp NYC dataset^(29)^. Food retailers were further categorised as supermarkets and bodegas if met criteria. Bodega and supermarket data were drawn from the NYCDOHMH^(28,29)^. The NYCDOHMH defined supermarkets as retail food stores of 10 000 square feet or greater, and/or food retailers with multiple chain locations regardless of size (such as Key Foods). Bodegas were defined by the NYCDOHMH as food stores that are less than 4000 square feet. Stores that appeared to be restricted to specialties or non-food items based on their title (such as smoke shops, meat markets and bakers) were excluded^(28,29)^. Finally, the five dimensions of food environment diversity were accounted for in our reporting of the findings according to the GeoFERN guidelines^(30)^.

### Covariates

In the final models, self-reported age in years (‘What is your age?)’, educational attainment (‘What is the highest grade or year of school you have completed?’), birthplace (‘Where you born in the U.S. or one of its territories’) and race (identifying as Black/African American, largest racial minoritised sub-group in study sample) were adjusted for and presented in this article.

### Statistical analysis

Addresses from each food environment and participant variable were geocoded into QGIS 3.14 using the MMQGIS ‘Geocode’ plug-in. Any address not found was reviewed and re-geocoded so that all available variables with legitimate addresses were input in QGIS 3.14^(31)^. Density of each food environment indicator was calculated by counting each variable in each community district and divided by each community’s area in square meters. Participant addresses were also geocoded in QGIS 3.14. A buffer at a standard buffer distance of 800 m (about a half mile or a 10–15-min walk) was drawn around each participant’s address using a Euclidean buffer approach. The rationale for such a cut-off includes participants’ walkability likelihood in the context of New York City (about a half mile or a 10–15-min walk) and as 0·5 miles represents a low-access point in urban areas as defined by USDA^(32)^. Each food environment indicator was counted within each buffer area. LPA was used to identify individuals’ residential urban food environment profiles, based on the quantity of each food environment indicator within each participant’s buffer. Using this approach, unobserved groups were classified into profiles based on dietary patterns. Three cluster structures were tested under the assumption that there would be 2-to-4 unique neighbourhood profile memberships in each cluster. Akaike information criterion (AIC) and the Bayesian information criterion (BIC) were used to assess model performance across cluster structures. Latent profiles were then visualised using QGIS 3.14. Group memberships based on these ‘profiles’ were also associated with observed dietary behaviour outcomes using LPA, accounting for covariates (age, foreign-born status and race). Associations between profile memberships and dietary outcomes were tested using mixed linear regression (i.e. multilevel model using reduced maximum likelihood). Listwise deletion was used to handle missing data and participants’ responses under ‘do not know’ or ‘refused’. Getis-Ord GI* local clusters were found for each LPA group using GeoDa, which were then visualised with kernel density heatmaps in QGIS 3.14.

## Results

### Participants characteristics and model fit statistics

**Fig. 1:**
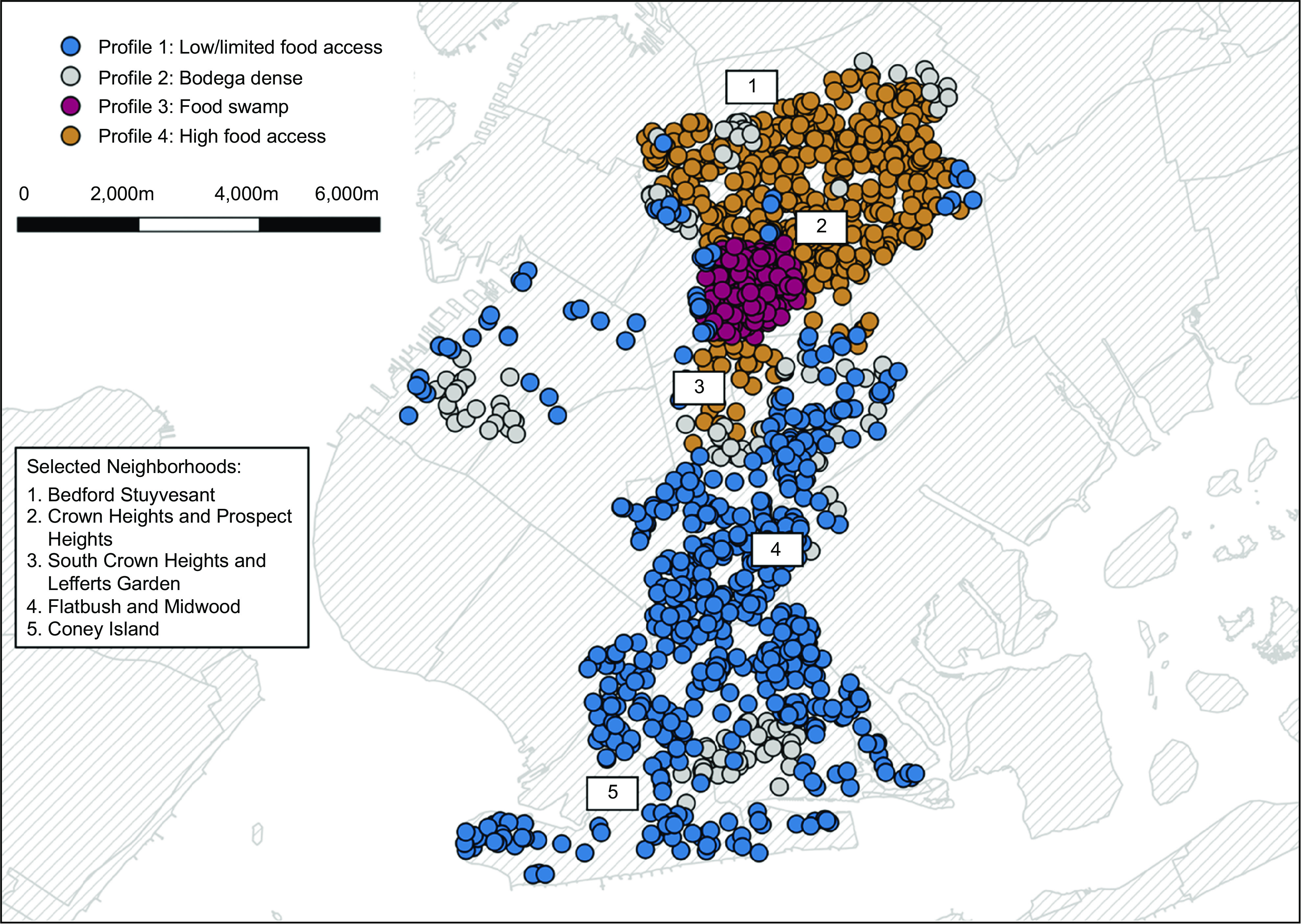
Participant addresses by profile; each point represents a survey participant’s home address. Each point in profile 1 (represented with blue) represents individuals in the limited/low access group. Profile 2 (light grey) is represented by individuals in the bodega-dense neighbourhoods. Profile 3 (pink) represents individuals in the food swamp group. Profile 4 (merigold) represents individuals in the high food access group

Among survey respondents, the mean age was 61 (sd = 12·89), 71 % of participants identified as female and 54 % participants identified as Black or African American. Thirty-eight percentage of participants were born outside of the USA, and LPA identified four residential urban food environment profiles (with significant high clusters ranging from 17 to 57 across profiles), as visualised in Fig. 1. The four-profile LPA model was the best fitting model with the lowest goodness of fit values compared with the two- (*AIC =* –2426·49; *BIC =* –2341·56) and three-profile models (*AIC =* –614·12; *BIC =* –571·65).

### Identification of residential urban food environment profiles

**Fig. 2:**
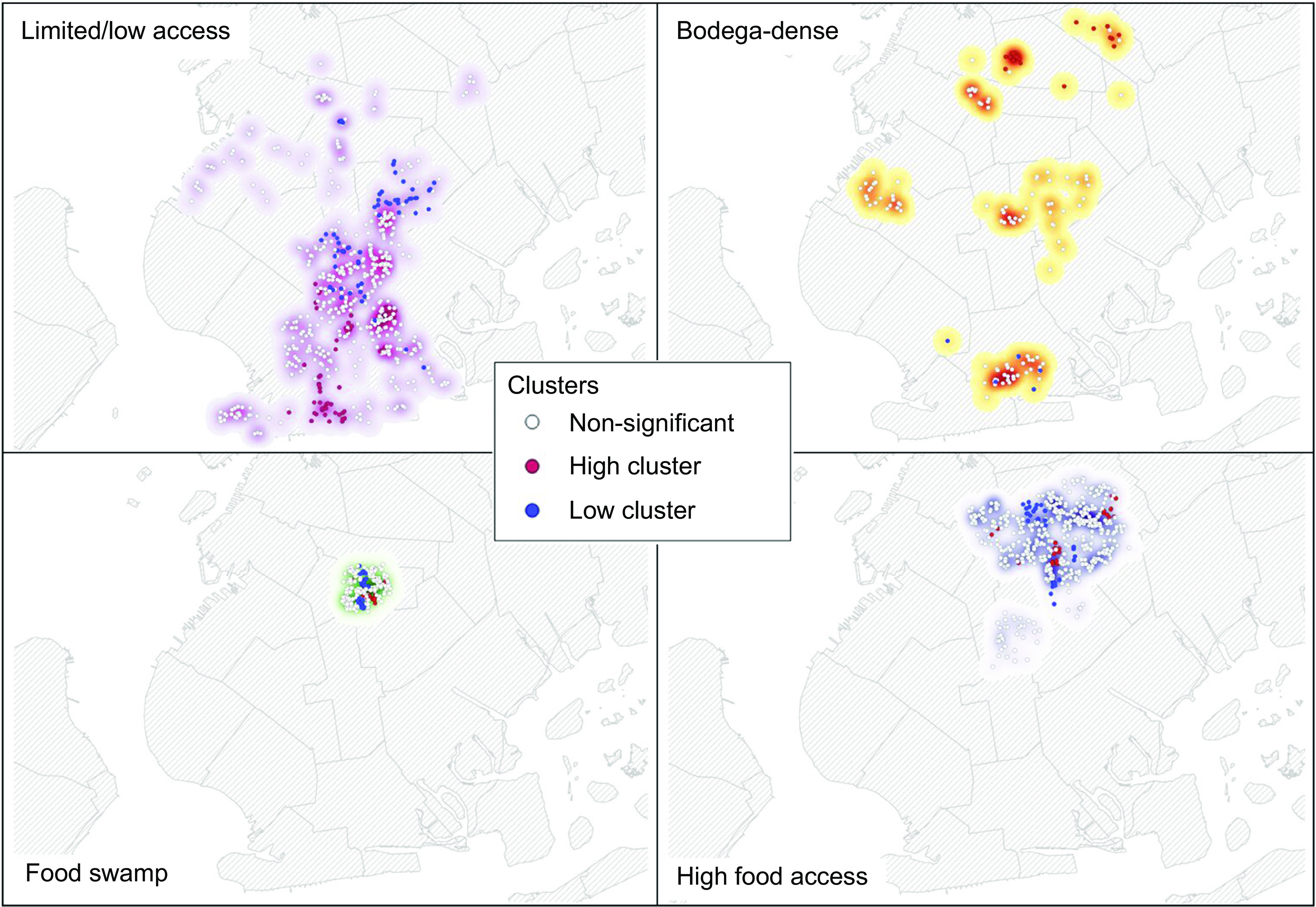
Kernel density heat profiles with high/low clusters across four residential urban food environment profiles: (a) limited low/access, (b) bodega-dense, (c) food swamp and (d) high food access. Each point represents a participant address. Red dots indicate high cluster significance; blue dots indicate low cluster significance. White dots indicate non-significance

The first residential profile termed the ‘limited/low food access’ group (*n* 587) was characterised by a likelihood of exposure on a few large supermarkets or restaurants, with much less likelihood to access nearby food pantries, community kitchens, farmer’s markets or bodegas, and overall, very low food access compared with other groups. In this profile, more than half (65 %) of residents self-identified as non-Hispanic White. In addition, over half of the sample in this profile had a college degree or postgraduate training (52·13 %). The second profile was the bodega-dense neighbourhood group (*n* 140), characterised by a likelihood of exposure to either large supermarkets or bodegas for food access and with some access to farmer’s markets. This profile has the least access to restaurants in their own communities and some access to SNAP sign-up locations and food pantries. Nearly half (46 %) of residents in this profile self-identified as Black or African American and the highest proportion of high school (26·43 %) and college graduates (27·14 %). A third profile, the food swamp group (*n* 254), was characterised by individuals likely relying on an abundance of nearby fast-food restaurants, with some access to bodegas, farmer’s markets and food pantries, and the lowest access to community kitchens and SNAP sign-up locations. Seventy-three percentage of this profile’s participants identify as Black or African American in comparison with 16 % who identify as non-Hispanic White. This profile also included the highest proportion of participants who identify as male (38·35 %). Finally, there is a high food access neighbourhood profile (*n* 512), characterised by moderate to high access of the majority of food outlets nearby but lower availability of restaurants nearby. The majority of participants (79 %) associated with this profile identify as Black or African American. Lastly, the highest proportion of participation in SNAP (16·21 %) and Supplemental Security Income (SSI) (10·74 %) were represented in this profile. Overall, the limited/low food access profile had fifty-seven significantly high clusters, bodega-dense neighbourhood profile had twenty-one significantly high clusters, food swamp profile had seventeen significantly high clusters and high food access profile had twenty-five significantly high clusters (Fig. 2).

### Association of residential urban food environment profiles and diet

Results from the mixed linear models can be found in Table 3. Dietary consumption of fruits and vegetables was not statistically different across the four residential urban food environment profiles identified. On average, participants in the limited/low food access profile were more likely to consume SSB compared with those in the bodega-dense profile (b = 0·44, *P* < 0·05) in adjusted models. Also, participants identifying as male were more likely to consume SSB (b = 0·48, *P* < 0·01). Participants who identified as Black or African American also were more likely to report greater SSB intake (b = 0·48, *P* < 0·01) than other racial and ethnic groups in this sample. Participants who were born in the USA were less likely to consume fruit (b = –0·15, *P* < 0·01) but more likely to consume SSB (b = 0·19, *P* < 0·01). Lastly, the relationship between educational attainment and consumption of fruit (b = 0·04, *P* < 0·01), vegetables (b = 0·09, *P* < 0·01) and SSB (b = –0·04, *P* < 0·05) was statistically significant.

**Table 2:** Demographic information of survey participants by residential urban food environment profile and average across all participants

		Profile 1: limited/low access (*n* 587)	Profile 2: bodega-dense (*n* 140)	Profile 3: food swamp (*n* 254)	Profile 4: high food access (*n* 512)	All participants (*n* 1493)
Age	Mean age	60·28	61·23	63·23	60·87	61·07
Identified sex (%)	Female	67·97	62·14	71·65	77·15	71·20
	Male	32·03	37·86	38·35	22·85	28·80
Race (%)	Black/African American	23·00	46·47	73·23	79·69	54·12
	Caucasian/White	65·07	31·43	16·14	8·01	34·03
	Hispanic/Latino	5·28	9·29	4·33	8·01	6·43
	Asian/Pacific Islander	4·43	2·14	2·76	1·37	2·88
	Native American/Indigenous	1·70	0·00	1·97	2·54	1·88
	Arab/Middle Eastern	0·51	0·00	0·39	0·59	0·47
	Other	4·60	4·29	9·45	3·32	4·96
Educational attainment (%)	Elementary/some high school	4·43	8·57	11·02	12·11	8·51
	High school graduate	21·81	26·43	24·02	26·37	24·18
	Tech, trade or vocational school	1·70	2·86	1·57	2·34	2·01
	Some college	19·59	13·57	23·23	21·48	20·29
	College graduate	26·41	27·14	22·44	20·31	23·71
	Postgraduate/professional school	25·72	20·00	17·32	16·80	20·70
Participation in federal safety nets (%)	SSI	8·01	8·57	10·62	10·74	9·44
	SNAP	7·16	12·14	13·38	16·21	11·79
	Free/reduced price lunch	2·39	0·71	0·40	2·73	2·21
	TANF	0·34	1·43	0·20	0·98	0·87
	WIC	0·68	0·00	0·39	1·17	0·80

SSI, Supplemental Security Income; SNAP, Supplemental Nutrition Assistance Program; TANF, Temporary Assistance for Needy Families; WIC, The Special Supplemental Nutrition Program for Women, Infants and Children.

**Table 3:** Multilevel model results for predicting diet outcomes from neighbourhood food profiles and socio-demographic factors using reduced maximum likelihood (REML)

	Fruit intake (servings/d)				Vegetable intake (servings/d)				SSB intake (servings/d)			
	B	se	Z	95 % CI	B	se	Z	95 % CI	B	se	Z	95 % CI
Variables												
Limited food access (ref)	–	–	–	–	–	–	–	–	–	–	–	–
Bodega-dense	−0·06	0·09	−0·63	−0·25, 0·12	−0·10	0·09	−10·06	−0·28, 0·08	−0·24*	0·10*	−2·29*	−0·45, –0·03
Food swamp	0·07	0·08	−0·80	−0·10, 0·24	0·05	0·07	0·75	−0·09, 0·21	0·06	0·09	0·63	−0·13, 0·25
High food access	−0·05	0·07	−0·77	−0·19, 0·08	−0·01	0·06	−0·17	−0·14, 0·12	0·13	0·07	10·70	−0·02, 0·29
Age	−0·00	0·00	−0·40	−0·00, 0·00	−0·00*	0·00*	−10·87*	−0·00, 0·00	0·00	0·00	10·21	−0·00, 0·00
Education	0·04**	0·01**	3·21**	0·01, 0·07	0·09**	0·01**	6·05**	0·06, 0·12	−0·04*	0·01*	−2·42*	−0·07, –0·00
Sex	−0·02	0·05	−0·48	−0·14, 0·08	−0·10	0·05	−1·91	−0·21, 0·00	0·48**	0·06**	7·62**	0·35, 0·60
NH Black	−0·03	0·06	−0·51	−0·15, 0·09	−0·10	0·06	−1·72	−0·22, 0·01	0·48**	0·06**	7·04**	−0·34, 0·61
US born	−0·15**	0·05**	−2·99**	−0·26, –0·05	0·09	0·05	1·71	−0·01, 0·19	0·19**	0·05**	3·26**	0·07, 0·30
Intercept	1·77**	0·16**	10·86**	1·45, 2·09	1·63**	0·16**	10·19**	1·32, 1·95	0·68**	0·18**	3·83**	0·33, 1·04

B, unstandardised coefficient; Z, standard score statistic.

* *P* < 0·05.

** *P* < 0·01.

## Discussion

Our results suggest that there was four distinct residential urban food environment profiles among a sample of adults residing in Brooklyn, New York in Spring 2020. These four profiles were the limited/low food access profile, the bodega-dense profile, food swamp profile and high food access profile. Measuring the local food environment is complex^(6)^, which often necessitates combining multiple environmental assessment techniques in addition to GIS-based measures. Nonetheless, our study reflects research in the literature describing food environment variability across multiple groups through clustering methods while accounting for participants’ socio-demographic characteristics^(13,19,33–35)^.

A study by Diehl and colleagues^(34)^ found that individuals in a social disadvantaged profile (i.e. categorised using stratified sex, race, ethnicity and income) were less likely to find healthy items at the closest grocery store compared with their counterparts in social advantaged groups. Studies by Berger and colleagues^(13)^ and Richardson *et al*.^(19)^ used a similar approach but with the ultimate goal of correlating BMI and access to unhealthy food outlets in the USA over time. They found that there was a strong correlation between BMI and the increase of unhealthy food retail stores (e.g. fast-food restaurants) over a 20-year span. One study combined both physical activity and food environment indicators when building their latent class/profile models^(33)^, which yielded three profiles in which at least two clustered similar characteristics to the profiles emergent in our study (i.e. low/limited access and high access groups). Lastly, a study in the cities of Geelong and Melbourne (Victoria, Australia) found three profiles with neighbourhood characteristics clustering in a slightly related fashion compared with our study. These three profiles were labelled ‘variety of outlets’, ‘café/restaurants & convenience’ and ‘few types of outlets’^(35)^.

Overall, improving on the precision of food environment measurement is warranted. Location and proximity are just a couple of important features out of a wider variety denoting the residential food environment. Additional inquiries in this area should expand beyond residential boundaries and also be able to assess individuals’ interaction with their food environment (i.e. activity space level)^(36,37)^. There is also a wide range of methodological differences across studies denoting the food environment, which differ in scale and shapes of buffers, study settings, among others^(16,38)^.

While we identified four distinct food environment profiles, we did not see the same association between the profiles and dietary patterns previously described in the literature. Instead, we found that participants in low or limited food access neighbourhood profiles were more likely to consume SSB compared with those in bodega-dense profiles, which deviates from recent studies suggesting bodegas are a common place to purchase low-nutritive value, caloric-dense food products such as SSB^(11,39)^. There are several plausible reasons our studies limited dietary associations with the identified profiles.

First, GIS-based food environment measures (albeit common) are the least consistently associated with diet in comparison to^(6)^ measures that rely on participant-reported or store audit data to assess dimensions of food access (i.e. perceived availability, affordability and quality). Participant-reported measures tend to range from single-item indicators to short scales^(6)^. Store audit measures were more comprehensive, on average, including the presence, price and quality of fresh produce and other food products. Lastly, participant-reported measures used in past studies seem to demonstrate higher degrees of reliability compared with store audit measures^(6)^.

Second, error in dietary data can affect our ability to make inferences regarding the association between residential urban food environment profiles and diet^(40)^. Social desirability bias (i.e. individuals reporting what they perceive as healthier diets compared with what they actually consume) can skew results to look like individuals eat healthier diets than they do. Lastly, a key source that can influence dietary measurement in this study was recall bias (i.e. individuals mis-remembering their food choices), which certainly impact the accuracy of the resulting dietary data collected. In the literature, diet- and health-related outcomes are measured in diverse ways leading to results that make comparisons across studies difficult and may explain mixed evidence in this field of study^(41,42)^.

Furthermore, the cross-sectional nature of the study design does not capture potential changes and differences in environment–behaviour interactions. In addition, food environment retail data only represent retailers registered with New York City open data sources at a single point in time and may not fully capture the changing neighbourhood food landscape. Furthermore, diet indicators examined (i.e. fruit, vegetables, SSB) may not fully capture the diet quality of individuals sampled. Lastly, the measurement used to assess buffer zone is considered a simple GIS-based measure even if there is not a ‘gold standard’ approach available. In using such approach, our inference might be limited as it does not consider food access that takes place outside of people’s own neighbourhoods but frequent often (i.e. near workplace or near school environments for those who are parents). We were also limited in making additional inferences about the study results since our models did not include data on all food retailers that may be present in New York City (i.e. supercentres, full-service restaurants, dollar stores, pharmacies, among others). Lastly, the adults in the study sample had a high average age and thus results may not be generalisable to a younger adult population.

In spite of these limitations, we had several findings that warrant further discussion. Our results revealed that participants identifying as Black and male were more likely to consume SSB and Black participants were less likely to consume vegetables. We found two food environment profiles (food swamp and high access) to be heavily populated by Black residents. While the above findings would be expected in a food swamp whereby a disproportionate number of unhealthy food options (dense in calories, high in Na and high in sugar) are sold by retailers that often use predatory marketing tactics^(43)^, we generally would not expect adults in a high food access environment to be at the same risk. Interestingly, in the current study, we observed that members of the high food access group also had the highest self-report of a member of the household receiving SNAP, SSI and WIC. The latter suggests a complicated interplay between the utilisation of federal food assistance benefits and access to one’s own local food environment to alleviate food insecurity. There is also research indicating that urban areas regardless of the neighbourhood often present the most challenges to access nutrient-dense foods for Black communities and individuals with low income^(44)^. Disparities in the residential food environments of New York City and Brooklyn in particular are driven by a myriad of factors, including racial and economic disparities, which impact access to affordable and/or health-promoting food choices^(45,46)^, and ultimately diet-associated chronic disease risk^(47)^.

Conceivably, findings have a bit more meaning where people are less likely to travel outside their residential neighbourhoods and leverage their residential urban food environments. Also, as it is documented that in the USA, individuals with low income used their local food environment more than those with greater socio-economic status^(37)^, survey participants in our study were oversampled to be respondents with low income^(25)^.

Future studies should investigate potential explanations for why individuals in limited and low food access neighbourhoods (e.g. food deserts) consume significant amounts of SSB. In addition to elucidating on food environment contributors to SSB intake, better understanding of drivers that may improve dietary choices could help shape evidence-based public health policies and programmes in urban settings. Our findings suggest increasing access to food retailers such as farmer’s markets, as characterised in the bodega-dense profile, could be an important safety net for urban residents with low income to make nutrient-dense food and beverage choices, especially if they are incentivised^(48)^. In addition, conducting a food store audit^(49)^ or considering consumer nutrition environmental factors (i.e. product price, quality, affordability and in store characteristics)^(50)^ would provide more information on other factors of the food retail environment that can be accounted for to improve the precision of food environment measurement. Lastly, the research team anticipates that these findings will inform subsequent efforts to identify multi-stakeholder strategies to promote nutrient-dense food access in local food environments among urban residents with low income and from minoritised backgrounds in counties and cities similar to Brooklyn.

## Conclusion

In sum, this study identified four residential urban food environment profiles in Brooklyn, New York. Individuals in limited and low food access neighbourhoods are in a susceptible position to consume significant amounts of SSB compared with those in bodega-dense neighbourhoods. Further research is warranted to elucidate strategies to improve fruits and vegetables consumption and reducing SSB intake within residential urban food environments.
